# Smoking Cessation Strategies After Acute Coronary Syndrome

**DOI:** 10.3390/jcm14041388

**Published:** 2025-02-19

**Authors:** Anum Nazir, Smrthi Shetty Ujjar, Moncef Oualid Seddiki, Mala Jheinga, Lampson Fan

**Affiliations:** Cardiothoracic Directorate, Heart and Lung Center, New Cross Hospital, Royal Wolverhampton Trust, Wolverhampton WV10 0QP, UK; anum.nazir@nhs.net (A.N.); smrthi.shetty@nhs.net (S.S.U.); moncef.seddiki@nhs.net (M.O.S.); mala.jheinga@nhs.net (M.J.)

**Keywords:** nicotine replacement therapy, varenicline, bupropion, cystisine, cognitive behavioural therapies

## Abstract

Smoking is one of the strongest modifiable risk factors for coronary artery disease. It is the cause of approximately 10–30% of deaths due to cardiovascular disease around the world. There is a 50% reduction in the risk of myocardial infarction by one year for people who successfully quit smoking. Considering the risk associated with smoking and the benefits of smoking cessation, it is important to identify and implement effective smoking cessation strategies. There are pharmacological as well as non-pharmacological interventions to assist in smoking cessation. Pharmacological therapies including nicotine replacement therapy; bupropion and varenicline have generally been studied more in patients with cardiovascular disease than the non-pharmacological interventions. Non-pharmacological strategies for smoking cessation include behavioural interventions such as counselling sessions and cognitive behavioural therapy. Studies and randomised controlled trials have demonstrated the safety of most of the pharmacological interventions. Nonetheless, the success rates are variable for the different pharmacological options. Data suggest that greater success can be achieved in smoking cessation with a combination of pharmacological and non-pharmacological treatment. However, more studies are needed to explore the best therapeutic options to improve the success of smoking cessation.

## 1. Introduction

### 1.1. Prevalence and Side Effects

Smoking is injurious to health, and it is an issue of worldwide concern. It reduces life expectancy on average by 10 years in smokers and is a cause of approximately eight million mortalities each year globally [1,2]. It further leads to a reduction of around 20 years in good health and well-being for smokers before death due to morbidity [3].

Smoking is linked to deleterious health effects and multiple pathologies. It is one of the strongest risk factors for cardiovascular disease including stroke, myocardial infarction, and peripheral arterial disease. Approximately 10–30% of deaths due to cardiovascular disease around the world are associated with smoking. The odds ratio for smokers in comparison to non-smokers who suffer from a myocardial infarction is ~2.5. Based on epidemiologic as well as scientific studies, it has been seen that smoking and cardiovascular effects have a non-linear trend. This means that even a low quantity of exposure to smoke is linked with an unduly high risk of cardiovascular disease. This trend also clarifies the reason why passive smokers carry a significant 25–30% increased risk of cardiovascular disease. It highlights the importance of advising patients with cardiovascular disease to try and avoid exposure to second-hand smoke [3,4].

According to the World Health Organization, approximately 22.3% of the global population was using tobacco in 2020. Tobacco is used most frequently in the form of cigarette smoking worldwide. There are around 1.3 billion users of tobacco worldwide and 80% of these reside in either low or middle-income countries [2]. In the United Kingdom, approximately 6.6 million adults are smokers, and this accounts for about 13.3% of the total adult population in the country. The incidence of smoking is proportionately linked to deprivation in the United Kingdom, with higher smoking numbers being witnessed among the unemployed population and people with mental health issues [1,5].

Cigarette smoke is known to contain approximately 4000 chemical compounds. The main toxic component of cigarette smoke is nicotine. However, other toxic components of cigarettes also have deleterious effects, and the main identified toxins include tar and gases. Tar forms the dry particulate matter of cigarette smoke. This particulate matter has many dangerous substances, including metals, dioxins, nitrosamines, and polynuclear aromatic hydrocarbons. These chemicals are carcinogenic and increase the risk of various cancers in the human body. Some toxins are also found in the form of gases, and these include carbon monoxide and benzene. Carbon monoxide has a higher affinity for haemoglobin than oxygen and forms a compound known as carboxyhaemoglobin. This leads to a reduction in the oxygen-carrying capacity of haemoglobin. Concentrations of 2 per cent or more of carboxyhaemoglobin result in reduced blood flow to the heart, leading to angina in patients with underlying cardiac disease [6,7]. 

### 1.2. Addiction to Nicotine

The main chemical in cigarette smoking which leads to addiction is nicotine. The definition of addiction is difficulty and limitation in stopping or reducing smoking over a prolonged time. To be categorized as a smoker, an individual should be actively smoking and should have smoked a minimum of 100 cigarettes in their entire life. Cigarette smoking can be referred to as a chronic condition that involves not only physical but also psychological dependence. The chemical substance nicotine mainly leads to the production of stimulating neurotransmitters which lead to a feeling of well-being and hence over time leads to physical dependence. Social along with environmental factors also tend to play a role and make smoking cessation even more challenging. An example of this is that some individuals are habituated to smoking in stress or like to smoke in social gatherings [3,8]. 

Nicotine acts mainly by binding on the cholinergic receptors of nicotine. These are found in the human body at the neuromuscular junction, in the adrenal glands, the autonomic ganglia, and the brain. The actions on the heart and the vasculature are mainly due to the sympathetic stimulation caused by nicotine. This is achieved by various mechanisms including central as well as peripheral stimulation of the sympathetic system. Central stimulation occurs mainly due to the effect of nicotine on peripheral chemoreceptor activation. Peripherally, the adrenals and the vascular nerve endings also tend to release higher levels of catecholamine in response to nicotine. Nicotine results in the increased production of dopamine, serotonin, nitric oxide, norepinephrine, epinephrine, and beta-endorphins. The increased release of catecholamines results in increased coronary vascular resistance. It also increases conduction through the atrioventricular node which may lead to arrhythmias. The abnormal release of nitric oxide due to nicotine also contributes to endothelial injury and hence is a contributing factor for atherogenesis [9]. 

### 1.3. Benefits of Smoking Cessation

The cessation of smoking provides an advantage in terms of health improvement irrespective of how long an individual has smoked or the number of cigarettes an individual has smoked in their lifetime [1,3]. Even if an individual quits smoking at the age of 60 years, this adds 3 years to their life and leads to a reduction in death from cancer and cardiac disease. For individuals who stop smoking before middle age, the life expectancy increases by 10 years, and the risk of early death by all causes is reduced [1,3,10]. A relative risk reduction of around 45% in mortality from cardiovascular diseases is noted with smoking cessation. This is higher compared to high-intensity statin and blood pressure control with a decrease of 10mmHg, as the relative risk reduction with these measures is around 35% and 15%, respectively. Despite this, approximately 50% of patients continue to smoke after their presentation with acute coronary syndrome [8].

The benefits of quitting smoking can be seen relatively soon following cessation. Within 20 min of smoking cessation, the heart rate of a person starts to normalize. In 3 days, improvement in the respiratory pathway is noted and carbon monoxide levels diminish in the body. The risk of myocardial infarction reduces to 50 percent by 1 year and it decreases to a similar level as a non-smoker in approximately 15 years [11].

Smoking also poses an economic burden not only due to the costs incurred on the healthcare sector to treat the diseases but also due to reduced productivity at the workplace, loss of income, and damage caused by fire. In 2012, it was estimated that the economic load due to smoking was around 1.8% of the global yearly gross domestic product [1].

**Figure 1:**
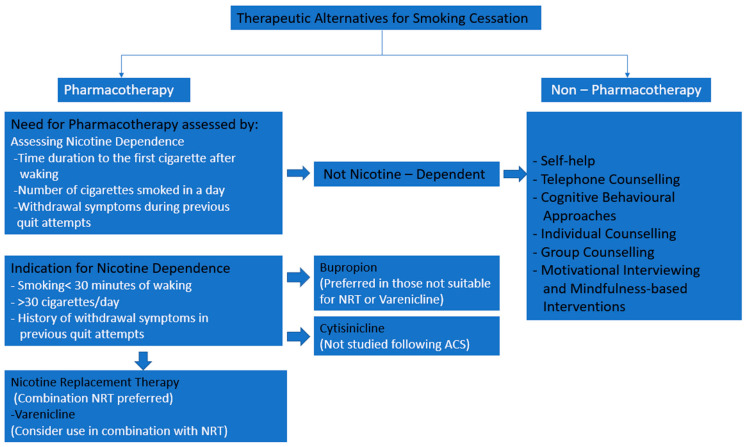
Approaches to smoking cessation.

Smoking is an important modifiable risk factor for coronary disease. Considering its significant association with cardiovascular morbidity and mortality, this is an important secondary prevention aspect that needs good focus and management strategies [3,8]. Various pharmacological and non-pharmacological intervention strategies are used for smoking cessation. Figure 1 shows the approach to smoking cessation. This review examines pharmacological strategies, including nicotine replacement therapy, bupropion, varenicline, and cytisine alongside non-pharmacological approaches such as cognitive behavioural therapy, counselling, and motivational interviewing. It explains in depth the benefits and mechanisms of action of the pharmacological therapies along with their safety profile. It further highlights the advantages and positive outcomes seen with using non-pharmacological therapy. 

## 2. Non-Pharmacological Treatment for Smoking Cessation

### 2.1. General Principles

Of the 40% of smokers who initiate a cessation attempt, only 2% achieve sustained abstinence. The initial two-day period is a particularly vulnerable time, as two-thirds of relapses occur during this timeframe [12]. Smoking, similar to a chronic condition, requires repeated interventions and long-term support to maintain abstinence [13]. Three common approaches to smoking cessation counselling include the 5As/5Rs, motivational interviewing, and the Stages of Change model. The 5As involve asking about tobacco use, advising to quit, assessing willingness to quit, assisting with treatment, and arranging follow-up. If unwilling to stop, the 5Rs focus on relevance, personalizing the risks of smoking, rewards of quitting, roadblocks, and repeating the advice [12].

### 2.2. Specific Interventions

Non-pharmacological interventions can be implemented through both clinical and public health strategies. Clinical approaches primarily include self-help programmes, telephone counselling, and cognitive behavioural therapies, which may be implemented for individual patients and as public health initiatives [13].

#### 2.2.1. Self-Help Programmes

While many smokers quit independently, structured programmes can improve cessation rates. This may include any printed self-help materials, such as booklets, leaflets, or information sheets, that offer a structured programme and guidance for smoking cessation [14]. Self-help programmes typically involve printed or electronic materials provided to patients to enhance their motivation to quit and offer guidance on achieving this goal. They offer several advantages: they are relatively cost-effective, the written materials can be reused for subsequent attempts to stop, these materials can be tailored for different target groups, and smokers can alter them to their individual needs [13].

A Cochrane review of 11 trials found no significant benefit for structured self-help materials without face-to-face contact compared to no treatment (n = 20,264; RR 1.06; 95% CI 0.95 to 1.18). However, 31 trials within the same review demonstrated that self-help materials tailored to individual smoker needs were more effective than standard materials compared to no treatment (n = 13,437; RR 1.35; 95% CI 1.19 to 1.53). Tailored self-help materials, designed to meet individual smoker needs, are more effective than standard materials, although the overall impact may still be modest [15].

#### 2.2.2. Telephone Counselling

Telephone counselling can complement face-to-face consultations or support self-help interventions and pharmacotherapy. It may facilitate smoking cessation or relapse prevention.

Counselling can be proactive, initiated by the counsellor, or reactive, provided on demand through helplines or quit lines [13,15].

A Cochrane review of 104 trials involving general population smokers found that those who contacted quit lines (n = 32,484; risk ratio (RR) 1.38; 95% confidence interval (CI) 1.19 to 1.61) had higher quit rates with multiple counselling sessions compared to self-help materials. In the same review, three additional trials found higher quit rates among smokers who received three to five telephone counselling calls compared to just one call (RR 1.27; 95% CI 1.12 to 1.44; 2602 participants) [15]. A Canadian hospital study (n = 276) involving patients with acute coronary syndrome or coronary artery bypass surgery (CABG) found higher abstinence rates (62%) in patients receiving the intensive intervention (bedside education, counselling, self-help materials, and 7-day nurse-led post-discharge telephone counselling) compared to minimal intervention (odds ratio [OR] 2.0; 95% confidence interval [CI] 1.2–3.1) [16].

#### 2.2.3. Cognitive Behavioural Approaches

Non-pharmacological therapeutic approaches, including cognitive behavioural and motivational therapies, may enhance smoking cessation outcomes by focusing on negative mood states, cravings, and habitual behaviours [13]. Psychological interventions include self-help materials, brief therapist-delivered interventions, intensive individual or group counselling, and a combination of different approaches [17]. Behavioural strategies address the pleasurable associations and triggers that maintain smoking, while cognitive strategies target thoughts and emotions related to smoking, such as perceived lack of control or stress relief. Cognitive behavioural therapy-based treatments are highly effective for quitting and preventing relapse [18].

#### 2.2.4. Individual Counselling

Individual counselling typically involves reviewing smoking history, identifying high-risk behaviours, and developing coping strategies. Counsellors may offer support, encouragement, and additional materials like written materials, videos, or audiotapes [17,19].

A systematic review of 49 trials with 19,000 participants demonstrated that individual counselling is more effective than minimal intervention along with offering no pharmacotherapy [17]. A randomized controlled trial (RCT) conducted in England with 540 patients admitted after myocardial infarction or for CABG showed that single-session intervention did not affect smoking cessation in chronic heavy smokers [20]. A meta-analysis of four RCTs showed that intensive behavioural therapy resulted in better smoking cessation [21].

#### 2.2.5. Group Counselling

Group behaviour therapy (usually four to eight) involves regular meetings to provide information, advice, and behavioural techniques. Group support can be beneficial, but there is limited evidence on whether it is more effective or cost-effective than individual counselling [19]. Group therapy provides an environment for learning behavioural techniques for smoking cessation and encouraging social support among participants [22].

A review article of 66 trials comparing group therapy with self-help, individual counselling, or no intervention demonstrated that group therapy improved cessation rates compared to self-help (n = 4395; risk ratio (RR) 1.88; 95% confidence interval (CI) 1.52 to 2.33) [23]. A review article of studies conducted in Asian countries revealed a paucity of evidence regarding the efficacy of group therapy for smoking cessation, despite its benefit in Western populations. More research is needed on group therapy for smoking cessation in Asian countries [24].

#### 2.2.6. Motivational Interviewing and Mindfulness-Based Interventions

Motivational interviewing is a widely used approach to facilitate smoking cessation by strengthening individual’s motivation and commitment to change their responses to smoking urges.

Motivational interviewing typically requires longer sessions than brief interventions and individual counselling. It is an evidence-based approach that helps smokers explore their uncertainty about quitting, set goals, and build self-motivation [22].

Mainstream behavioural treatments focus on avoiding triggers, managing stress, distracting from cravings, and building social support. However, triggers are common, and diverting attention can be difficult, especially after strong negative emotions. Additionally, finding healthy substitutes is not always easy. Given the role of emotions and cravings in smoking and the limitations of current treatments, innovative approaches are needed [25]. Newer treatments focus on managing negative emotions and cravings, rather than avoidance or substitution, using techniques like “urge surfing”. Recent research emphasizes recognizing and tolerating negative emotions [26]. Thus, mindfulness training, by targeting both feelings and cravings, may help people quit smoking. Mindfulness involves paying attention to the present moment with acceptance. It can help people become aware of their habits and the emotional triggers associated with smoking [27]. Mindfulness-based therapies include mindfulness training, acceptance and commitment therapy (ACT), and distress tolerance training. It involves meditation practice, while ACT and distress tolerance focus on accepting thoughts and feelings and developing coping strategies [28].

A recent Cochrane review found that mindfulness-based treatments may help improve emotional regulation, but there is no conclusive evidence that they help people quit smoking. More research is needed to determine whether mindfulness-based interventions can be effective for smoking cessation [28]. 

#### 2.2.7. Healthcare Provider Interventions

Opportunistic interventions by healthcare providers, such as brief advice and guideline-based programmes, can significantly reduce smoking rates. Recommending exercise as a complementary strategy can further enhance cessation efforts. Healthcare providers, including doctors, nurses, and pharmacists, can play a crucial role in helping patients quit smoking. Primary care providers are well positioned to intervene, and current guidelines strongly support this [13].

#### 2.2.8. Broad Dissemination/Public Health Approaches

Broad dissemination programmes target large populations through community, workplace, and media interventions. These programmes often use self-help, counselling, and healthcare provider interventions on a larger scale [29].

#### 2.2.9. Electronic Vaping Cigarettes (e-Cigarettes)

While modified risk products (MRPs) like e-cigarettes are often promoted as less harmful alternatives to traditional cigarettes, they still pose health risks. MRP use can release ultrafine particles, which are associated with increased cardiovascular complications, including myocardial infarction [30]. Although a network component meta-analysis of pharmacological and e-cigarette interventions demonstrated that nicotine e-cigarettes increased abstinence rates [31], another systemic review and meta-analysis showed that the existing evidence of cardiovascular safety is concerning. There is a need for further studies to address these concerns before marketing them as a safe alternative to traditional cigarettes [32].

## 3. Pharmacological Interventions

### 3.1. Nicotine Replacement Therapy

Treatment with nicotine replacement therapy is based on the principle that there is measured nicotine intake in the form of replacement which assists in refraining from tobacco and smoking. This pharmacological treatment helps in overcoming the symptoms of withdrawal associated with smoking cessation as the controlled release of nicotine from nicotine replacement therapy works by stimulating the receptors of nicotine. The absorption of nicotine is slower this way owing to the pharmacokinetics of nicotine replacement therapy. There are various types of nicotine replacement options available. This includes a longer-acting option which are transdermal patches. The short-acting options are nicotine gums, inhalers, and lozenges. A combination of short- and long-acting nicotine replacement therapy tends to show better results. The contraindication to the use of nicotine replacement therapy is mainly allergy, and there are no other significant reasons to avoid using it. Based on multiple controlled and case–controlled trials, nicotine replacement therapy does not lead to a higher number of cardiovascular events; however, an increase in tachycardia and arrhythmia is noted due to the positive effects of nicotine on the sympathetic system [3,33].

The instant effects on the cardiovascular system due to nicotine are a transient rise in the heart rate by around 10–15 beats in a minute and a rise in the blood pressure, mainly systolic, up to a maximum of 10 mmHg [34]. However, many studies have now demonstrated that these effects of nicotine do not increase the risk of cardiovascular events in patients with coronary artery disease. There are two important studies for patients with stable coronary artery disease which showed that there was no rise in the risk of cardiovascular events in the subgroup that was receiving nicotine replacement therapy. This included The Working Group for the Study of Transdermal Nicotine in Patients with Coronary Artery Disease (n = 156) and a study by Joseph et al. (n = 584) [34,35]. Another retrospective study by Dhaliwal et al. in 2024 compared patients admitted with acute coronary syndrome who received nicotine replacement therapy with patients who did not receive nicotine replacement therapy. It showed that there is no significant increased risk of adverse cardiovascular events in patients admitted with acute coronary syndrome who are prescribed nicotine replacement therapy. This study was based on the comparison of 122,198 patients who were given nicotine replacement therapy with 160,387 patients who did not receive it. The patients who received nicotine replacement therapy had lower chances of cardiovascular events as compared to patients who did not receive nicotine replacement therapy. This included a reduced risk of arrhythmia, cardiac arrest, and mortality [36]. 

Based on their meta-analysis of 24 trials exploring smoking cessation success in patients who received pharmacological and behavioural interventions, Suissa et al. suggested in 2017 that no conclusive evidence for the efficacy of nicotine replacement therapy could be seen (RR: 1.22; 95% CI 0.72–2.06) [37]. However, a Cochrane-based data collection and analysis based on 82 studies concluded with a high certainty of evidence that nicotine replacement therapy assisted a significant number of patients to quit smoking [38]. Furthermore, based on 68 completed studies, Theodoulou et al. suggested in 2023 that there is a high degree of evidence that combinations of nicotine replacement therapies are superior to single forms of nicotine replacement therapy used for patients [39].

### 3.2. Bupropion

Bupropion is an atypical antidepressant functioning as a weak selective dopamine and noradrenaline reuptake inhibitor [40]. It was first introduced in 1974 as a treatment for depression and seasonal affective disorder and was later licenced for smoking cessation [41]. Although its mechanism of action is not fully understood, it is proposed that by inhibiting the reuptake of dopamine and noradrenaline, it disrupts the reward pathways associated with nicotine use, reducing craving and withdrawal effects [3].

#### Efficacy of Bupropion in Smoking Cessation

Bupropion is an effective medication for smoking cessation. The rate of abstinence after 12 months of treatment was doubled in bupropion users compared to placebo and was shown to be equally effective in both sexes [3,42].

A systematic review by Suissa et al. in 2017 found bupropion to be beneficial in smoking cessation in patients with stable cardiovascular disease (CVD) compared to placebo (RR: 2.46; 95% CI 1.63–3.71), but that it was of small or no benefit in acute CVD (RR: 1.16; 95% CI 0.90–1.50) [37,43].

Bupropion is safe and generally well tolerated. The most common side effects are dry mouth, insomnia, headache, nausea, and vomiting [40,44]. Rarely, bupropion has been associated with seizures (1:1000). The risk is higher in individuals with certain predisposing factors such as a history of seizure disorders, central nervous system (CNS) tumour, or concurrent use of medications that lower the seizure threshold [3]. Concerns have previously been raised about potential neuropsychiatric adverse effects associated with bupropion. However, the EAGLES study (2016) demonstrated no significant increase in neuropsychiatric events with bupropion compared to placebo [37,45].

In individuals with stable CVD, studies have not identified any significant safety concerns related to bupropion use [3]. Furthermore, evidence suggests that bupropion can be safely administered even in the setting of acute CVD, with no reported adverse cardiovascular effects when initiated immediately following an acute CVD event [46].

Bupropion as a modified release (MR) tablet is licenced and recommended as a pharmacological treatment for smoking cessation and is recommended as part of the pharmacotherapy for smoking cessation post-myocardial infarction [47,48]. Therapy starts 7–14 days before the decided smoking cessation date, for a total of 7–9 weeks. The dose is started at 150 mg once a day and increased to 150 mg twice daily after 6 days of treatment. Caution is required in elderly patients or cases of renal or hepatic impairment.

### 3.3. Varenicline 

Varenicline is an α4β2 nicotinic acetylcholine receptor (nAChRs) partial agonist, designed for smoking cessation. Its partial activation of the α4β2 receptor (which is related to dopamine release following nicotine binding) alleviates symptoms of craving and withdrawal during abstinence whilst also reducing the reinforcing effects of nicotine by preventing binding [3,49].

There has been an increasing number of randomised controlled trials (RCTs) and systematic reviews evaluating the use of varenicline, which have shown it to be a valuable therapy for smoking cessation and superior when compared to alternatives [50,51,52]. Varenicline was first introduced in the UK in 2006 (as Champix) but later withdrawn in 2021 after concerns that it contained above-acceptable nitrosamine levels. However, it was licenced and reintroduced in August 2024 with compliant levels and Medicines and Healthcare products Regulatory Agency (MHRA) safety approval. It is recommended as a first-line treatment for tobacco dependence [53,54].

Many studies have found varenicline to be effective in smoking cessation when used as monotherapy or in combination. Cahill et al. found varenicline can increase the chance of abstinence from smoking at 6 months by 2–3 times compared with placebo [55]. A 2022 Cochrane review suggested moderate-certainty evidence that varenicline was more effective in smoking cessation in hospitalized patients than placebo or no pharmacotherapy (RR 1.29; 95% CI 0.96 to 1.75; four studies; 829 participants) [38]. This is further supported by Wu et al. who, after analysing four trials, found that varenicline was superior to placebo treatment even one year later [56]. Wu, along with other studies, showed that varenicline was more effective than bupropion [38,57,58,59] and nicotine replacement therapy monotherapies [59,60]. 

Suissa et al.’s network meta-analysis studied several therapies in participants with cardiovascular disease. It showed that varenicline was associated with greater abstinence than other pharmacological and behavioural treatments [37]. Guo et al.’s network meta-analysis also identified varenicline as superior to counselling, but no significant difference was found in smoking cessation compared with cytisine (OR = 0.93; 95% CI [0.62, 1.39]) [60].

Regarding combined interventions, Guo et al. showed that varenicline combined with behavioural (SMS and counselling), nicotine replacement therapy, or bupropion was more effective in cessation than placebo, bupropion, nicotine replacement therapy, and counselling alone, but this was not significantly different from varenicline monotherapy. Varenicline + bupropion was considered the best intervention, which may be superior to varenicline + nicotine replacement therapy (OR = 1.21; 95% CI [0.74, 1.96]) [60]. Several meta-analyses support the varenicline plus bupropion combination as being first-ranked for abstinence [59], whilst others differ. In a 2015 systematic review and meta-analysis, Chang et al. indicated that combination therapy with varenicline and nicotine replacement therapy was superior to varenicline monotherapy [61]. Vogeler et al. and Zhong et al. suggested varenicline combined with behavioural therapy may have a better outcome, and Stead et al. also reported the benefit of combined pharmacotherapy and behavioural treatment [62,63,64]. Opposingly, the results of Guo et al. showed that varenicline combined with behavioural therapy was not associated with high or certain evidence for smoking cessation [60].

The EVITA (Evaluation of Varenicline in Smoking Cessation for Patients Post-Acute Coronary Syndrome) trial examined the efficacy of varenicline for smoking cessation in patients hospitalised with ACS, enabling the evaluation of smokers with cardiovascular disease. The trial found that those randomised to varenicline had significantly higher rates of smoking abstinence and reduction than those randomised to placebo. At 12 months, the point-prevalence abstinence rates were 47.3% in the varenicline group and 32.5% in the placebo group (*p* = 0.012), the continuous abstinence rates were 35.8% and 25.8%, respectively (*p* = 0.081), and the rates of reduction ≥50% in daily cigarette consumption were 67.4% and 55.6%, respectively (*p* = 0.05) [65].

The use of Varenicline has raised safety concerns regarding the potential increased risk of cardiovascular events including myocardial infarction (MI) and stroke [66,67]. Singh et al. published a meta-analysis in 2011 suggesting that patients receiving varenicline had a numerically but not statistically greater rate of serious adverse cardiovascular events than those receiving a placebo (1.06% vs. 0.82%; OR 1.72; 95% CI 1.09–2.71) [66]. This study was subject to scrutiny and criticism due to several methodological concerns, such as the exclusion of studies in which no cardiovascular events were stated [49,65]. In the following year, Prochaska and Hilton published a meta-analysis, which found no increase in cardiovascular risk with varenicline (risk difference 0.27%; 95% CI −0.10 to 0.63), the findings of which were supported by an FDA-mandated meta-analysis suggesting no statistically significant association (incidence of cardiovascular mortality: varenicline 0.05%, placebo 0.07%; all-cause mortality: varenicline 0.14%, placebo 0.25%) [49,68].

Although the EVITA trial is not adequately designed to assess the safety of varenicline in patients after ACS, the population included has been the highest risk involved in any trial to date. Over 90% of enrolled patients had an MI within a few days of commencing treatment. No significant differences in either adverse event rates or major adverse cardiovascular event rates were found (serious adverse events: varenicline 11.9%, placebo 11.3%; major adverse cardiovascular events: varenicline 4.0%, placebo 4.6%). However, out of 39 serious adverse events reported within 30 days of treatment discontinuation, there were 2 deaths, 6 myocardial infarctions, and 5 cases of unstable angina [65]. Given the two deaths that occurred, further scrutiny of ACS patients is warranted. The most common side effects are nausea, headaches, and insomnia [54,65]. Others include abdominal pain, constipation, dizziness, dry mouth, weight increase, and increased appetite [3,54,65]. These tend to occur in the first 4 weeks of treatment, are temporary, and are mild to moderately severe [3]. 

There has been increased interest surrounding the incidence of neuropsychiatric side effects of both varenicline and bupropion, including depression, suicide and suicidal ideation, and the worsening of pre-existing psychiatric conditions [49]. Results from the 2016 Evaluating the Safety and Efficacy of Varenicline and Bupropion for Smoking Cessation in Patients With and Without a History of Psychiatric Disorders (EAGLES) trial showed no significant increase in neuropsychiatric adverse events associated with varenicline or bupropion compared with placebo or nicotine replacement therapy [45]. This is supported by data from numerous other studies that suggest that there is no increased risk of serious neuropsychiatric events when taking varenicline [3,49,55,69,70]. 

### 3.4. Cytisinicline (Cytisine)

Cytisine is derived from the Cytisus laburnum plant which partially activates the αlpha4 βeta2 nicotinic acetylcholine receptor and is responsible for nicotine’s central effects. This receptor subtype is linked to nicotine addiction and is the primary target of varenicline [71].

Cytisine was developed by Bulgarian pharmacologists in the early 1950s but was not widely used. Cytisine has been available in Central and Eastern Europe since the 1970s. Poland remains the main market for over-the-counter cytisine, contributing significantly to global tobacco control efforts, and until recently, cytisine was not available in the UK [72]. The standard treatment course is 25 days. Evidence suggests that 12 weeks of treatment will likely be more effective than the 25-day treatment [73]. Oral cytisine reaches peak levels in two hours. It is excreted unchanged by the kidneys and has a shorter half-life (4.8 vs. 17 h) and treatment course (3.5 vs. 12 weeks) than varenicline [74]. The main side effects of cytisine are gastric problems and sleep disturbances [75]. It is contraindicated in patients who have had recent myocardial infarction, unstable angina, serious arrhythmias, stroke, and women who are pregnant or breastfeeding. It has been advised to be used cautiously in patients with ischemic heart disease, hypertension, heart failure, and diabetes. It is not recommended in patients with renal or hepatic impairment and those over 65 years of age [72].

**Figure 2:**
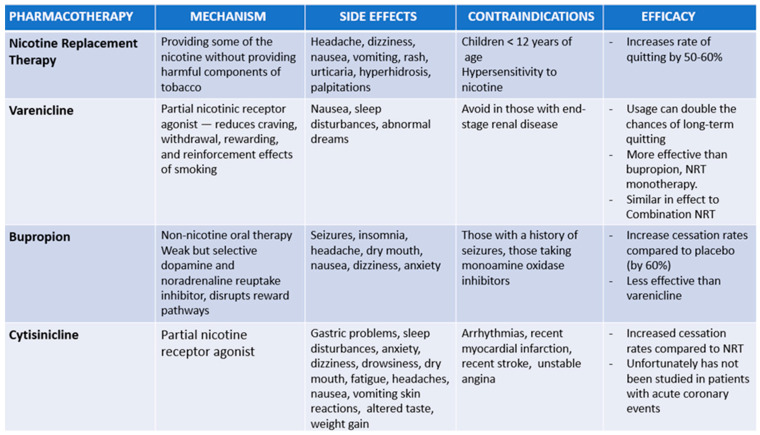
Pharmacological therapies for smoking cessation.

A Cochrane meta-analysis showed smoking cessation rates compared to control among cytisine users (OR 2.21; 95% CrI 1.66 to 2.97; seven RCTs; 3848 participants) [31]. A trial conducted in New Zealand to assess the efficacy of cytisine compared to nicotine replacement therapy demonstrated that cytisine was superior in maintaining abstinence [76]. A randomized controlled trial comparing 6-week and 12-week durations of cytisine therapy, combined with behavioural support, against placebo demonstrated favourable cessation rates and tolerability [77]. Another trial conducted in New Zealand comparing 12 weeks of cytisine treatment with varenicline showed that cytisine was at least as effective as varenicline in maintaining abstinence at 6 months [12]. Unfortunately, no trial has been performed to study the effect of cytisine following an acute coronary event. Figure 2 summarises the different pharmacotherapy options for smoking cessation. Table 1 shows the studies done to demonstrate the efficacy of pharmacological and behavioural therapy in patients with acute coronary syndrome.

## 4. Conclusions

The management of acute coronary syndrome has seen significant advancements in the past few decades. However, smoking cessation, which is an extremely beneficial secondary preventive measure, has not been given the much-needed attention. Most of the patients admitted with acute coronary syndrome have very low smoking cessation rates, which can contribute to their morbidity and mortality. Smoking cessation counselling initiated in the hospital can result in increased chances of quitting. Non-pharmacological interventions combined with pharmacotherapy can enhance quitting rates post-discharge. Data suggest that varenicline and bupropion are effective smoking cessation medications in patients with stable coronary artery disease. Bupropion is a valuable tool for smoking cessation in patients with stable cardiovascular disease, offering demonstrated efficacy, safety profile, and a non-nicotine mechanism of action. However, its role in acute coronary syndrome remains mixed. Similarly, there is a need for further study on the efficacy and cardiovascular safety of varenicline in the setting of acute coronary syndrome despite the EVITA trial showing promising results. Cytisine, another partial nicotine receptor agonist shown to be beneficial, has not been studied in patients post-ACS and is contraindicated in these patients. To conclude, there are effective pharmacological and non-pharmacological options for smoking cessation; however, given the paucity of data, there is a need for continued research to study the safety of medications in patients admitted with acute coronary syndrome.

## Figures and Tables

**Table 1:** Efficacy of pharmacological and behavioural therapy for smoking cessation in patients with acute coronary syndrome.

Study (Year)	THERAPY	n	Duration of Treatment	PPA (6 mo)	CA (12 mo)	LTF (n)
Eisenberg et al. [65] (2016)	Varenicline + MCI Placebo + MCI	151 151	Varenicline 0.5 mg QD × 3 days, BD × 4 days, and 1.0 mg BD for 11 weeks	47.3% 32.5%	31.1% 21.2%	45 37
Smith & Burgees [16] (2009) ∞	Intensive intervention M I	137 139	Bedside counselling, self-help, 7 tele counselling sessions at 2, 7, 14, 21, 30, 45 and 60 days	66.7% 48.9	54% 35%	12 13
Rigotti et al. [46] (2006)	Bupropion + IC	127	Bupropion CR 150 mg QD × 3 days and BD × 9 weeks	-	26%	39
Tonstad et al. [42] (2003)	Bupropion Placebo	629	Bupropion SR 150 mg BD × 7 weeks	-	22% 9%	17 19

PPA—Point prevalence abstinence; CA—Continuous abstinence; BD—Twice a day; QD—Once a day; LTF—Lost to follow up; 6 mo—6 months; 12 mo—12 months; MCI—Minimal clinical inter vention; MI—Minimal intervention (advice from doctors and pamphlets); IC—Intensive counselling. ∞—77.4% and 74.1% of the study participants in the intervention and control group included ACS patients.

## References

[1] Hartmann-Boyce J., Livingstone-Banks J., Ordóñez-Mena J.M., Fanshawe T.R., Lindson N., Freeman S.C., Sutton A.J., Theodoulou A., Aveyard P. (2021). Behavioural interventions for smoking cessation: An overview and network meta-analysis. Cochrane Database Syst. Rev..

[2] Tobacco. n.d. https://www.who.int/news-room/fact-sheets/detail/tobacco.

[3] Giulietti F., Filipponi A., Rosettani G., Giordano P., Iacoacci C., Spannella F., Sarzani R. (2020). Pharmacological Approach to Smoking Cessation: An Updated Review for Daily Clinical Practice. High Blood Press. Cardiovasc. Prev..

[4] Abroug H., El Hraiech A., Mehrez O., Fredj M.B., Zemni I., Salah A.B., Azaiez M., Jomaa W., Maatouk F., Belguith A. (2020). Acute coronary syndrome: Factors predicting smoking cessation. East. Mediterr. Health J..

[5] Smoking Cessation: What Is the Prevalence of Smoking? n.d. https://cks.nice.org.uk/topics/smoking-cessation.

[6] Bates M.N. The Chemical Constituents in Cigarettes and Cigarette Smoke: Priorities for Harm Reduction. https://www.researchgate.net/publication/265540805.

[7] Smith C.J., Hansch C. (2000). The relative toxicity of compounds in mainstream cigarette smoke condensate. Food Chem. Toxicol..

[8] McCaughey C.J., Murphy G., Jones J., Mirza K.B., Hensey M. (2023). Safety and efficacy of e-cigarettes in those with atherosclerotic disease: A review. Open Heart.

[9] Benowitz N.L., Gourlay S.G. (1997). Cardiovascular Toxicity of Nicotine: Implications for Nicotine Replacement Therapy 11All editorial decisions for this article, including selection of referees, were made by a Guest Editor. This policy applies to all articles with authors from the University of California San Francisco. J. Am. Coll. Cardiol..

[10] Doll R., Peto R., Boreham J., Sutherland I. (2004). Mortality in relation to smoking: 50 years’ observations on male British doctors. BMJ.

[11] Benefits of Stopping Smoking|Background Information|Smoking Cessation|CKS|NICE. n.d. https://cks.nice.org.uk/topics/smoking-cessation/background-information/benefits-of-stopping-smoking/.

[12] Hughes J.R. (2003). Motivating and helping smokers to stop smoking. J. Gen. Intern. Med..

[13] Niaura R. (2008). Nonpharmacologic Therapy for Smoking Cessation: Characteristics and Efficacy of Current Approaches. Am. J. Med..

[14] Livingstone-Banks J., Ordóñez-Mena J.M., Hartmann-Boyce J. (2019). Print-based self-help interventions for smoking cessation. Cochrane Database Syst. Rev..

[15] Matkin W., Ordóñez-Mena J.M., Hartmann-Boyce J. (2019). Telephone counselling for smoking cessation. Cochrane Database Syst. Rev..

[16] Smith P.M., Burgess E. (2009). Smoking cessation initiated during hospital stay for patients with coronary artery disease: A randomized controlled trial. Can. Med. Assoc. J..

[17] Lancaster T., Stead L.F. (2017). Individual behavioural counselling for smoking cessation. Cochrane Database Syst. Rev..

[18] Perkins K.A., Conklin C.A., Levine M.D. (2013). Cognitive-Behavioral Therapy for Smoking Cessation a Practical Guidebook to the Most Effective Treatments.

[19] RACGP—Supporting Smoking Cessation: A Guide for Health Professionals. n.d. https://www.racgp.org.au/clinical-resources/clinical-guidelines/key-racgp-guidelines/view-all-racgp-guidelines/supporting-smoking-cessation.

[20] Hajek P., Taylor T.Z., Mills P. (2002). Brief intervention during hospital admission to help patients to give up smoking after myocardial infarction and bypass surgery: Randomised controlled trial. BMJ.

[21] Mottillo S., Filion K.B., Bélisle P., Joseph L., Gervais A., O’Loughlin J., Paradis G., Pihl R., Pilote L., Rinfret S. (2009). Behavioural interventions for smoking cessation: A meta-analysis of randomized controlled trials. Eur. Heart J..

[22] 7.15 Individual and Group-Based Cessation Assistance—Tobacco in Australia. n.d. https://www.tobaccoinaustralia.org.au/chapter-7-cessation/7-15-methods-services-and-products-for-quitting-mo.

[23] Stead L.F., Carroll A.J., Lancaster T. (2017). Group behaviour therapy programmes for smoking cessation. Cochrane Database Syst. Rev..

[24] Mohamed R., Bullen C., Hairi F.M., Nordin A.S.A. (2021). A systematic review of group therapy programs for smoking cessation in Asian countries. Tob. Induc. Dis..

[25] Brewer J.A., Mallik S., Babuscio T.A., Nich C., Johnson H.E., Deleone C.M., Minnix-Cotton C.A., Byrne S.A., Kober H., Weinstein A.J. (2011). Mindfulness training for smoking cessation: Results from a randomized controlled trial. Drug Alcohol. Depend..

[26] Marlatt A.G., Donovan D.M. (2005). Relapse Prevention: Maintenance Strategies in the Treatment of Addictive Behaviors.

[27] Brewer J.A., Sinha R., Chen J.A., Michalsen R.N., Babuscio T.A., Nich C., Grier A., Bergquist K.L., Reis D.L., Potenza M.N. (2009). Mindfulness Training and Stress Reactivity in Substance Abuse: Results from a Randomized, Controlled Stage I Pilot Study. Subst. Abus..

[28] Jackson S., Brown J., Norris E., Livingstone-Banks J., Hayes E., Lindson N. (2022). Mindfulness for smoking cessation. Cochrane Database Syst. Rev..

[29] Hyland A., Li Q., Bauer J., Giovino G., Steger C., Cummings K.M. (2004). Predictors of cessation in a cohort of current and former smokers followed over 13 years. Nicotine Tob. Res..

[30] Alzahrani T., Pena I., Temesgen N., Glantz S.A. (2018). Association Between Electronic Cigarette Use and Myocardial Infarction. Am. J. Prev. Med..

[31] Lindson N., Theodoulou A., Ordóñez-Mena J.M., Fanshawe T.R., Sutton A.J., Livingstone-Banks J., Hajizadeh A., Zhu S., Aveyard P., Freeman S.C. (2023). Pharmacological and electronic cigarette interventions for smoking cessation in adults: Component network meta-analyses. Cochrane Database Syst. Rev..

[32] Siddiqi T.J., Rashid A.M., Siddiqi A.K., Anwer A., Usman M.S., Sakhi H., Bhatnagar A., Hamburg N.M., Hirsch G.A., Rodriguez C.J. (2023). Association of Electronic Cigarette Exposure on Cardiovascular Health: A Systematic Review and Meta-Analysis. Curr. Probl. Cardiol..

[33] Chehab O.M., Dakik H.A. (2018). Interventions for smoking cessation in patients admitted with Acute Coronary Syndrome: A review. Postgrad. Med. J..

[34] Pack Q.R., Priya A., Lagu T.C., Pekow P.S., Atreya A., Rigotti N.A., Lindenauer P.K. (2018). Short-Term Safety of Nicotine Replacement in Smokers Hospitalized with Coronary Heart Disease. J. Am. Heart Assoc..

[35] Ford C.L., Zlabek J.A. (2005). Nicotine Replacement Therapy and Cardiovascular Disease. Mayo Clin. Proc..

[36] Dhaliwal J., Sekhon M., Rajotia A., Ramphul K., Singh S. (2024). Abstract 4146448: Real-world Outcomes of Nicotine Replacement Therapy in Acute Coronary Syndrome. Circulation.

[37] Suissa K., Larivière J., Eisenberg M.J., Eberg M., Gore G.C., Grad R., Joseph L., Reynier P.M., Filion K.B. (2017). Efficacy and Safety of Smoking Cessation Interventions in Patients with Cardiovascular Disease. Circ. Cardiovasc. Qual. Outcomes.

[38] Rigotti N.A., Clair C., Munafo M.R., Stead L.F. (2024). Interventions for smoking cessation in hospitalised patients. Cochrane Database Syst. Rev..

[39] Theodoulou A., Chepkin S.C., Ye W., Fanshawe T.R., Bullen C., Hartmann-Boyce J., Livingstone-Banks J., Hajizadeh A., Lindson N. (2023). Different doses, durations and modes of delivery of nicotine replacement therapy for smoking cessation. Cochrane Database Syst. Rev..

[40] Bupropion|Prescribing Information|Smoking Cessation|CKS|NICE. n.d. https://cks.nice.org.uk/topics/smoking-cessation/prescribing-information/bupropion/.

[41] Huecker M.R., Smiley A., Saadabadi A. (2025). Bupropion. StatPearls.

[42] Tonstad S. (2003). Bupropion SR for smoking cessation in smokers with cardiovascular disease: A multicentre, randomised study. Eur. Heart J..

[43] Franck C., Filion K.B., Eisenberg M.J. (2018). Smoking Cessation in Patients with Acute Coronary Syndrome. Am. J. Cardiol..

[44] Wilkes S. (2008). The use of bupropion SR in cigarette smoking cessation. Int. J. Chron. Obs. Pulmon Dis..

[45] Anthenelli R.M., Benowitz N.L., West R., St Aubin L., McRae T., Lawrence D., Ascher J., Russ C., Krishen A., Evins A.E. (2016). Neuropsychiatric safety and efficacy of varenicline, bupropion, and nicotine patch in smokers with and without psychiatric disorders (EAGLES): A double-blind, randomised, placebo-controlled clinical trial. Lancet.

[46] Rigotti N.A., Thorndike A.N., Regan S., McKool K., Pasternak R.C., Chang Y., Swartz S., Torres-Finnerty N., Emmons K.M., Singer D.E. (2006). Bupropion for Smokers Hospitalized with Acute Cardiovascular Disease. Am. J. Med..

[47] (2021). Tobacco: Preventing Uptake, Promoting Quitting and Treating Dependence.

[48] Bupropion—StatPearls—NCBI Bookshelf. n.d. https://www.ncbi.nlm.nih.gov/books/NBK470212/.

[49] Windle S.B., Bata I., Madan M., Abramson B.L., Eisenberg M.J. (2015). A randomized controlled trial of the efficacy and safety of varenicline for smoking cessation after acute coronary syndrome: Design and methods of the Evaluation of Varenicline in Smoking Cessation for Patients Post-Acute Coronary Syndrome trial. Am. Heart J..

[50] Hughes J.R., Stead L.F., Hartmann-Boyce J., Cahill K., Lancaster T. (2014). Antidepressants for smoking cessation. Cochrane Database Syst. Rev..

[51] Livingstone-Banks J., Fanshawe T.R., Thomas K.H., Theodoulou A., Hajizadeh A., Hartman L., Lindson N. (2023). Nicotine receptor partial agonists for smoking cessation. Cochrane Database Syst. Rev..

[52] Jorenby D.E. (2006). Efficacy of Varenicline, an α4β2 Nicotinic Acetylcholine Receptor Partial Agonist, vs Placebo or Sustained-Release Bupropion for Smoking Cessation: A Randomized Controlled Trial. JAMA.

[53] Varenicline. n.d. https://www.ncsct.co.uk/publications/category/varenicline.

[54] Varenicline|Prescribing Information|Smoking Cessation|CKS|NICE. n.d. https://cks.nice.org.uk/topics/smoking-cessation/prescribing-information/varenicline/.

[55] Cahill K., Stevens S., Perera R., Lancaster T. (2013). Pharmacological interventions for smoking cessation: An overview and network meta-analysis. Cochrane Database Syst. Rev..

[56] Wu P., Wilson K., Dimoulas P., Mills E.J. (2006). Effectiveness of smoking cessation therapies: A systematic review and meta-analysis. BMC Public Health.

[57] Howes S., Hartmann-Boyce J., Livingstone-Banks J., Hong B., Lindson N. (2020). Antidepressants for smoking cessation. Cochrane Database Syst. Rev..

[58] Thomas K.H., Dalili M.N., López-López J.A., Keeney E., Phillippo D.M., Munafò M.R., Stevenson M., Caldwell D.M., Welton N.J. (2021). Smoking cessation medicines and e-cigarettes: A systematic review, network meta-analysis and cost-effectiveness analysis. Health Technol. Assess..

[59] Siskind D.J., Wu B.T., Wong T.T., Firth J., Kisely S. (2020). Pharmacological interventions for smoking cessation among people with schizophrenia spectrum disorders: A systematic review, meta-analysis, and network meta-analysis. Lancet Psychiatry.

[60] Guo K., Zhou L., Shang X., Yang C., Fenfen E., Wang Y., Xu M., Wu Y., Li Y., Li M. (2022). Varenicline and related interventions on smoking cessation: A systematic review and network meta-analysis. Drug Alcohol. Depend..

[61] Chang P.-H., Chiang C.-H., Ho W.-C., Wu P.-Z., Tsai J.-S., Guo F.-R. (2015). Combination therapy of varenicline with nicotine replacement therapy is better than varenicline alone: A systematic review and meta-analysis of randomized controlled trials. BMC Public Health.

[62] Vogeler T., McClain C., Evoy K.E. (2016). Combination bupropion SR and varenicline for smoking cessation: A systematic review. Am. J. Drug Alcohol. Abus..

[63] Zhong Z., Zhao S., Zhao Y., Xia S. (2019). Combination therapy of varenicline and bupropion in smoking cessation: A meta-analysis of the randomized controlled trials. Compr. Psychiatry.

[64] Stead L.F., Koilpillai P., Fanshawe T.R., Lancaster T. (2016). Combined pharmacotherapy and behavioural interventions for smoking cessation. Cochrane Database Syst. Rev..

[65] Eisenberg M.J., Windle S.B., Roy N., Old W., Grondin F.R., Bata I., Iskander A., Lauzon C., Srivastava N., Clarke A. (2016). Varenicline for Smoking Cessation in Hospitalized Patients with Acute Coronary Syndrome. Circulation.

[66] Singh S., Loke Y.K., Spangler J.G., Furberg C.D. (2011). Risk of serious adverse cardiovascular events associated with varenicline: A systematic review and meta-analysis. Can. Med. Assoc. J..

[67] Planer D., Lev I., Elitzur Y., Sharon N., Ouzan E., Pugatsch T., Chasid M., Rom M., Lotan C. (2011). Bupropion for Smoking Cessation in Patients with Acute Coronary Syndrome. Arch. Intern. Med..

[68] Ware J.H., Vetrovec G.W., Miller A.B., Van Tosh A., Gaffney M., Yunis C., Arteaga C., Borer J.S. (2013). Cardiovascular Safety of Varenicline. Am. J. Ther..

[69] Tonstad S., Davies S., Flammer M., Russ C., Hughes J. (2010). Psychiatric Adverse Events in Randomized, Double-Blind, Placebo-Controlled Clinical Trials of Varenicline. Drug Saf..

[70] Thomas K.H., Martin R.M., Knipe D.W., Higgins J.P.T., Gunnell D. (2015). Risk of neuropsychiatric adverse events associated with varenicline: Systematic review and meta-analysis. BMJ.

[71] Karnieg T., Wang X. (2018). Cytisine for smoking cessation. Can. Med. Assoc. J..

[72] Cytisinicline (Cytisine). n.d. https://www.ncsct.co.uk/publications/Cytisine-SPC.

[73] Jeong S.H., Newcombe D., Sheridan J., Tingle M. (2015). Pharmacokinetics of cytisine, an α 4 β 2 nicotinic receptor partial agonist, in healthy smokers following a single dose. Drug Test. Anal..

[74] Cahill K., Lindson-Hawley N., Thomas K.H., Fanshawe T.R., Lancaster T. (2016). Nicotine receptor partial agonists for smoking cessation. Cochrane Database Syst. Rev..

[75] Tutka P., Vinnikov D., Courtney R.J., Benowitz N.L. (2019). Cytisine for nicotine addiction treatment: A review of pharmacology, therapeutics and an update of clinical trial evidence for smoking cessation. Addiction.

[76] Walker N., Howe C., Glover M., McRobbie H., Barnes J., Nosa V., Parag V., Bassett B., Bullen C. (2014). Cytisine versus Nicotine for Smoking Cessation. N. Engl. J. Med..

[77] Rigotti N.A., Benowitz N.L., Prochaska J., Leischow S., Nides M., Blumenstein B., Clarke A., Cain D., Jacobs C. (2023). Cytisinicline for Smoking Cessation. JAMA.

